# Difficulties in Breast Feeding

**Published:** 1916-04

**Authors:** Mary Cleveland Harper

**Affiliations:** San Antonio, Texas


### Difficulties in Breast Feeding.

MARY CLEVELAND HARPER, M. D., SAN ANTONIO, TEXAS.

That there are difficulties in breast feeding will be admitted by many physicians and testified to by many mothers. That it is usually the first-born who is the greatest sufferer suggests the reason, ignorance—inexperience. Many young mothers have never even held a baby in their arms before their own was placed there. They must therefore look to the physician for advice and guidance, or become dependent on the neighborhood oracle, who may or may not lead her safely through the difficulties which confront her.

That the present-day mother does not nurse her baby because she does not wish to do so is not a fact in my experience. On the contrary, some of them are so jealous of this privilege that it is hard to convince them of the need for, and get their consent to, supplementary feeding, where the breast milk is not furnishing sufficient nourishment for the infant.

Very rarely it happens that a woman secretes no milk, when of course there is nothing to do but get a wet-nurse or put the infant on artificial food. I know of but three case of this sort, in two of which it is apparently an inherited condition. Fortunately the large majority of women functionate properly in this respect, at least in the early weeks of the life of the infant. One often hears a mother say: "I had plenty of milk at first, but it just gradually dried up, and I had to put the baby on the bottle." Unfortunately, another reason one hears for the "bottle baby" is that "the doctor advised me to wean the baby because my milk didn't seem to agree," or "because my milk poisoned the baby." Such advice is very seldom needed because such cases are really exceedingly rare.

To deprive an infant of its natural food without just cause is little short of criminal. In the first place, breast milk has been shown to possess certain immunizing properties which protect a child against disease; second, there is the difficulty of getting an artificial food which is uncontaminated—of getting the proper modification—of selecting a food which best meets the needs of the infant; and, third, artificial food is much more expensive, not only in the first cost of the food itself, but in treatment of the numerous digestive disturbances that follow in the train of artificial feeding. Our statistics show that the number of deaths from intestinal diseases, in babies under two years of age, exceeds the number of deaths of people of all ages, children and adults, from all of the contagions—measles, whooping cough, scarlet fever, diphtheria, meningitis and typhoid. It is further shown by statistics that for every breast-fed baby who dies there are six deaths of babies artificially fed.

It therefore behooves the members of our profession to very seriously consider the matter from every standpoint before advising a mother to wean her baby, and to use every means at our command to improve her supply of milk and to correct the digestive disturbances in the nursing babe.

The causes of poor milk supply are varied and may be divided into (1) pre-natal and (2) post-natal.

*Pre-natal.*—Under this head comes everything in the woman's life previous to the birth of the baby which would have a bearing on her own normal development, both physically and mentally,—good food, fresh air, suitable exercise, sufficient rest and sleep.

*Post-natal.*—Under this head comes everything which has any bearing on her physical well-being and peace of mind during the period of lactation. Particularly in the case of a primipara is it desirable to protect her from all undue anxiety as to the welfare of her baby. There are certain things which may come up during the early days of nursing which cause intense physical pain and mental worry, which, with proper care, may be avoided or at least ameliorated to a considerable degree. Among these is:

*The Fissured Nipple.*—This very painful condition may be prevented if, for a period of six or eight weeks preceding delivery, an astringent lotion, such as boric acid dr. 1, tannic acid, dr. 1, glycerine dr. 3, alcohol dr. 4, rose water, ad. qs. ounces 2, is applied to the nipples three times daily. This has a tendency to harden the nipples and prevent excoriations and fissuring after nursing begins. If, however, such condition should develop, nursing on the affected side should be temporarily discontinued, the breast milked regularly and massaged gently to prevent caking, using a little olive oil as a lubricant. I have found the promptest results to follow painting the nipples with a 10 per cent solution of silver nitrate once or twice daily. The breast should be protected with a sterile dressing to prevent infection. Mastitis is usually preceded by an excoriated nipple.

*The inverted or the abnormally small nipple* is another difficulty which disappears in most cases with proper care. The best way to promptly develop such a nipple is to put a strong, healthy baby to the breast. Half a dozen vigorous nursings will work wonders. As it is not always convenient to borrow a baby for this purpose, the nipple should be manipulated with the fingers for a few minutes before each nursing, or a breast pump or nipple shield used to draw out the nipple.

Where the baby cannot nurse because of weakness, or deformity of the sucking apparatus, the breast may be kept functionating by expressing the milk from the breast at regular intervals—say, every four hours—emptying it as completely as possible each time. From two to four ounces of milk may be obtained at each nursing in this way, and used in place of artificial food, greatly to the advantage of the baby.

The foregoing conditions may be considered as purely mechanical difficulties, and their prompt solution means the preservation of the natural food supply of the baby. The physical pain produced by the baby tugging on a fissured nipple, and the mental anxiety over the outcome of the other two conditions, are often sufficient to alter the quality and quantity of the milk to such an extent as to render it unfit for use. Indeed, an appreciation of the psychic element as a means of influencing woman's milk will sometimes give the solution to otherwise obscure digestive disturbances in a nursling.

*Example.*—About half an hour before nursing time, Mrs. S. received a telegram from her husband, announcing the death of a very dear friend of the family. The two-months-old baby was nursed at the regular time, and very promptly vomited the entire feeding. There was no fever and no temperature, but the nausea seemed to continue for as much as an hour afterwards. The mother was instructed to make no attempt to feed the baby until the next period, and in the meantime to give as much water as the baby would take. Four hours later the baby was again put to the breast and took a full feeding without any bad effect, and has continued to thrive ever since. This little patient had never been sick a minute in her life up to the time in question, had taken no food of any character except her mother's milk. She has continued perfectly well ever since.

There is no doubt but that the mental distress this woman suffered on hearing the news of her friend's death caused some alteration in the character of the milk which produced the nausea and vomiting in the baby.

*Example No. 2.*—The mother, a perfectly healthy woman, had nursed two previous children for nine months each. Her third baby was nursed successfully for four months. Just at this time the family had serious financial reverses, which was the source of much anxiety to the mother. Dating from this period of worry, the baby developed intestinal disturbance, vomiting and diarrhea, which did not cease until the child was weaned, some two months later. No further trouble occurred after the baby was put on a diet of modified milk. In this case, the mother was entirely unable to control her anxiety, in consequence of which a train of difficulties developed—loss of appetite, sleepless nights, alteration of the breast milk, digestive disturbances in the baby.

These are not unusual cases at all, and doubtless occur in the experience of every physician who practices on nursing babies.

I think it is generally agreed that rest, mental and physical—freedom from excitement, work and anxiety, with plenty of sleep, are essential to the production of good milk; that it is also influenced by the amount and character of exercise; that there should be at least one free bowel evacuation daily, and that the question of diet is of great importance. I think perhaps the diet is one of the most frequent causes of trouble. Some neighborhood "health expert" frightens the young mother with a long list of food which she cannot eat, telling her of the dire consequences following its use. Or perhaps she will be told to drink beer or other alcoholic beverage when plain water is indicated. Tea is another drink which is recommended, the use of which produces constipation in many women, a trouble which is so often present any way after pregnancy.

As a rule, I allow a nursing mother to eat practically anything that agrees with her. If her food is agreeing with her, it will be evidenced by an absence of such symptoms as gas, constipation, diarrhea, etc. I also encourage the mother to watch the symptoms that may develop in the baby following the ingestion of certain foods by the mother, in this way often being able to eliminate the offending article. There are certain things that seem to disagree with many nursing mothers, such as cabbage, sweet potatoes, salads, etc. Highly seasoned foods are undesirable also.

If the milk is too rich in fat or proteids, I recommend an increase of the water intake, taking as much as two quarts daily, cutting out all malt and alcoholic beverages, reducing the amount of meat and eggs, and exercise, preferably in the open air, and never to the point of exhaustion. Sometimes exercise alone will correct the trouble. If the milk is deficient in these elements—fat and proteid—take the articles of diet above mentioned, and plenty of rest. If the amount of milk is small, gruels and milk will be of service in increasing the supply.

I have also had some degree of success in increasing the milk supply by the use of Bier's pump for the purpose of producing a hyperemia and consequent stimulation of the gland. I apply the pump three or four times daily for a period of five to ten minutes at a time.

Where there is an insufficient supply of milk to properly nourish the baby, and the mother worries over its failure to gain in weight, thereby aggravating the condition, I do not hesitate to supply the shortage in food by suitable modification of cow's milk, giving this after each nursing instead of alternating the breast and bottle. This is often only a very temporary arrangement, for when the mother finds that the baby is gaining properly her milk improves to such an extent that it furnishes all the nutrition necessary. This is an experience I have had repeatedly.

There is another condition which sometimes causes trouble in the early days of nursing and which should be prevented. During the first two or three days of life, it is not an uncommon thing to find the infant plied with sweetened water every time it cries. The degree of sweetening varies with the taste of the nurse. There are some babies who escape such treatment without injury. There are many more who are seriously upset by it. I have seen vomiting, colic and diarrhea of a severe character follow it. There would be six to ten thin, watery, sour stools in the twenty-four hours, with excoriated buttocks as a natural consequence.

Personally, I believe plain boiled water, eight to sixteen ounces in the twenty-four hours, will answer every purpose for the first two or three days. Certainly a weak solution of condensed milk (one level teaspoonful to four ounces of boiled water), one to two ounces every three hours, will provide all the food necessary until the mother's milk begins to flow.

Because of the inability of the mother to read the signs of failure of her effort to nurse her baby, many a child has traveled so far along the road to starvation that it is with difficulty that it is started on an artificial food.

The signs of unsuccessful nursing are:

1. Very little gain, stationary, or loss in weight.
2. The baby cries and frets while at the breast and prolongs the nursing unduly. It may be satisfied temporarily, but begins to fret long before the next feeding period.
3. Where the quality, rather than the quantity of the milk is at fault, there may be abnormal stools—either constipation or diarrhea. They may also contain mucus and curd.
4. Baby does not sleep well, either night or day.
5. Vomiting.
6. Colic.

Where either one or all of the above conditions exist, it is well to inquire into the mother's habits of life, the manner of nursing the baby, particularly as to the length of nursings and the period between. If the probable cause of the trouble is not revealed by this inquiry, an examination of the milk is advisable. The fat and protein should be estimated and a microscopic examination also made.

In collecting the milk for this examination, all of the milk from one breast should be taken in order to get a fair average of the fat and protein. The first milk is poor and the last part may be very rich; at any rate, it is always richer than that which comes first. In making the microscopic examination, milk from both breasts should be taken. This is for the purpose of determining the presence of pus cells.

A case in point is the following: Baby four months old who had recurrent attacks of vomiting and diarrhea. Every effort was made to determine the cause. The milk from both breasts had been examined and found to be approximately normal. During treatment, he was taken from the breast from time to time, given a small dose of castor oil, put on barley or rice water, and in twenty-four to forty-eight hours was well. He was returned to the breast, nursed for five or six days, and another attack developed. The milk was finally examined microscopically and a considerable quantity of pus found to be present in the milk of the right breast. There was no pain, redness or swelling in the breast to indicate the presence of pus, but inquiry into the past history developed the fact that the right breast had been incised in the second week after the birth of the baby for the purpose of draining an abscess. This baby was weaned, and the intestinal disturbances promptly ceased.

*Contra-indications for nursing:*

1. *In any wasting disease,* such as nephritis, tuberculosis, or any malignant disease.

2. *In any acute disease* which will probably be of long duration, such as typhoid, pneumonia, etc. It is not necessary to discontinue nursing on account of malaria unless the disease is of a severe type and has produced a profound secondary anemia.

3. Where there is *mental disturbance,* or where the mother is so hopelessly neurotic that the quality and quantity of the milk varies from one nursing to the next, and produces constant symptoms of indigestion in the infant.

4. *During pregnancy.* A woman ought not to be expected to provide for a child in utero and a child in the arms at the same time. Besides, there are profound changes which occur in the body during pregnancy which may affect the nursing baby.

There are certain conditions under which the breast should be temporarily withheld. For instance:

1. *During menstruation,* provided any symptoms develop in the baby during that period. Some babies continue nursing at this time without any inconvenience, while others have loss of appetite, nausea, vomiting and diarrhea. This does not always last through the entire menstrual period, disappearing after the second or third day. In such cases, I discontinue the nursing until the third day and then put the baby to the breast again.

2. In acute vomiting of the baby, as in the cases above described, where there was some change produced in the mother's milk from distress and anxiety; or where the condition may have been produced by the ingestion of some improper food by the baby, as often happens, particularly where there are older children to feed them.

3. Where there is acute intestinal trouble from the same causes mentioned in the preceding paragraph.

4. Where the mother is acutely ill.

5. Where the mother is suffering severe mental or nervous distress; where she is overheated or physically exhausted.

*Examples.*—Nausea, vomiting and diarrhea coming on acutely within an hour after nursing, occurred in a little patient three months old, who, up to that time, had been perfectly well. The mother, a splendid specimen of womanhood, had come to San Antonio from a much cooler climate. The day of the baby's illness the mother had been on a shopping expedition, and remained beyond the baby's feeding hour. She rushed home, and, while very hot and tired, nursed her baby, with the result described above. She was instructed to omit the next feeding, giving plain boiled water, to which was added a few grains of sodium bicarbonate. The baby was put to the breast at the following nursing period without any bad symptoms appearing. The diarrhea ceased after eight or ten hours, and the baby had no further trouble.

If this woman had omitted the first feeding, given the baby a bottle of artificial food, milked out her breasts, and waited till the next feeding time to put her to the breast, the chances are there would have been no trouble.

6. *Abscess of the Breast.* In this condition, it may be necessary to permanently take the child from the affected breast, but this is not always so. Before allowing the child to take the breast again, the milk should be examined microscopically.

### CAUSES OF VOMITING IN THE BREAST-FED BABY.

1. The quality of the milk. The alteration in the milk may be due to something the mother has eaten, by worry, excitement, fatigue or being overheated, as indicated by examples given above. It may be due to an excess of fat in the milk.

2. It may be due to bad practice in feeding; that is, the baby is nursed irregularly or too frequently. This in itself has a bad effect on the milk. The breast that is emptied too frequently produces a milk high in fats. A stomach into which food is put before the previous meal has had time to be disposed of is certain to suffer distress. Fat is the element in milk which causes the most trouble and is slower in passing into the intestine.

3. Nursing a menstruating mother.

4. Nursing a pregnant mother.

### CAUSES OF CONSTIPATION IN BREAST-FED BABY.

1. Milk that is too high in fat will produce constipation in some children, while in others it will cause a diarrhea. It is often very difficult to convince a mother of this, as they are told that by adding cream to the diet of a bottle-baby constipation will be overcome. This unfortunately is not always true. Where constipation is produced by an excess of fat, the stool will often look like putty, to which a good deal of oil has been added, giving it a slick, shiny look; there will be a particularly foul odor. Sometimes they are dry and crumbly, but still of the putty color.

2. Insufficient food, through the failure to provide sufficient residue to stimulate peristalsis, will produce constipation. This may be corrected by giving supplementary feedings of modified cow's milk to babies under six months. To a healthy, well-developed baby of six months or older, one feeding of very thoroughly cooked cream of wheat or farina may be given. These should be cooked at least two hours, and the initial feeding made small, a level tablespoonful, to which a piece of butter the size of a filbert, or an ounce or two of cow's milk may be added. The milk should be clean, fresh milk, and it is always safer to have it boiled three to five minutes.

3. A constipated mother will almost invariably have a constipated baby, and hence a colicky baby. This is one of the hardest conditions that a physician has to contend with. There is usually a history of prolonged constipation in the mother, for which a formidable list of laxatives and purgatives have been used. Diet, exercise and an effort to form regular habit in the matter of evacuating the bowels are ordered for the mother, and in eight cases out of ten she proceeds exactly as before, preferring to resort to medication and enemas. I have found the mineral oils to be very beneficial in some cases, and have no effect in others. In selecting a laxative in these cases, it is always desirable to avoid the hydragogues. Two or three watery stools in twenty-four hours will appreciably decrease the amount of milk.

In conclusion, the nursing mother is entitled to the thoughtful care and consideration of every member of her household. She should be spared every worry, anxiety and fatigue possible, both for her own sake and for the little one who is dependent on her for sustenance. When the physician is consulted, he should spend all the time necessary to discover the cause of the trouble for which he was consulted, and of its satisfactory solution. Successful management of breast feeding, no less than bottle feeding, depends on an infinite capacity for attention to details.

---

At a recent meeting of the Texas State Board of Medical Examiners, Dr. S. L. Scothorn, of Dallas, was elected president; Dr. H. C. Morrow, of Austin, was elected vice president, and Dr. J. S. McCelvey, of Temple, was elected Secretary.

